# Observational study on medication administration errors at a University Hospital in Brazil: incidence, nature and associated factors

**DOI:** 10.1186/s40545-022-00443-x

**Published:** 2022-08-22

**Authors:** Lindemberg Assunção-Costa, Ivellise Costa de Sousa, Renata Kelly Rodrigues Silva, Ana Carla do Vale, Charleston Ribeiro Pinto, Juliana Ferreira Fernandes Machado, Cleidenete Gomes Valli, Luis Eugenio Portela Fernandes de Souza

**Affiliations:** 1 School of Pharmacy, Federal University of Bahia, Salvador, Bahia Brazil; 2 Hospital Universitário Professor Edgard Santos (HUPES/EBSERH), Salvador, Bahia Brazil; 3 Hospital Santo Antônio, Salvador, Bahia Brazil; 4 National Institute for Pharmaceutical Assistance and Pharmacoeconomics, Salvador, Bahia Brazil; 5 Health Department of the State of Bahia, Salvador, Bahia Brazil; 6 Institute of Collective Health, Federal University of Bahia, Salvador, Bahia Brazil; 7 Rua Alameda Salvador, 1057, Torre América, Sala 308, Caminho das árvores, 41820790 Salvador, Bahia Brazil

**Keywords:** Medication error, Hospital, Observational study, Direct observation, Patient safety, Medication systems

## Abstract

**Background:**

Medication administration errors are frequent and cause significant harm globally. However, only a few data are available on their prevalence, nature, and severity in developing countries, particularly in Brazil. This study attempts to determine the incidence, nature, and factors associated with medication administration errors observed in a university hospital.

**Methods:**

This was a prospective observational study, conducted in a clinical and surgical unit of a University Hospital in Brazil. Two previously trained professionals directly observed medication preparation and administration for 15 days, 24 h a day, in February 2020. The type of error, the category of the medication involved, according to the anatomical therapeutic chemical classification system, and associated risk factors were analyzed. Multivariate logistic regression was adopted to identify factors associated with errors.

**Results:**

The administration of 561 drug doses was observed. The mean total medication administration error rate was 36.2% (95% confidence interval 32.3–40.2). The main factors associated with time errors were interruptions. Regarding technique errors, the primary factors observed were the route of administration, interruptions, and workload.

**Conclusions:**

Here, we identified a high total medication administration error rate, the most frequent being technique, wrong time, dose, and omission errors. The factors associated with errors were interruptions, route of administration and workload, which agrees well with the results of other national and international studies.

**Supplementary Information:**

The online version contains supplementary material available at 10.1186/s40545-022-00443-x.

## Background

Medication errors in hospitals are frequent and can cause harm. The social and economic impacts of this challenge are already well known in developed countries, and, more recently, are being described in developing countries [1–4]. In 2017, the World Health Organization (WHO) launched the third global patient safety challenge, entitled “medication without harm”, with a bold goal of reducing harm caused by medication errors by 50%. Medication errors can occur at any stage of the medication process: prescription, dispensing, and administration of medicines, with the administration stage presenting the greatest risk, as it is the final stage before reaching the patient. To achieve the goal set by the WHO, it is necessary to obtain epidemiological data on the occurrence of errors, including those related to associated factors and the severity of their consequences, considering that most medication errors do not cause harm to the patient [5].

Little is known about the prevalence, nature, associated factors, and severity of errors in developing countries, particularly in Latin America. A recent systematic review, which included studies with the direct observation technique, identified a drug administration error rate of 32% (16–35.8% interquartile range) in this region, with high variability in prevalence (9–64%); in addition, only one study assessed the factors associated with medication administration errors (MAEs) and none assessed the severity of these errors [6]. A few studies in Brazil have adopted direct observation as the gold standard for estimating the rate of MAE. These studies differ widely in terms of their inclusion/exclusion criteria, definitions, and categorization of errors. Moreover, among all the Brazilian studies included in the aforementioned systematic review, only one described how to calculate the error rate [7].

Accordingly, this study aims to fill this knowledge gap by identifying the prevalence and nature of medication errors, including the associated factors, in a public university hospital in Brazil.

## Methods

### Study design and location

This is a prospective observational study that adopts the technique of direct-disguised observation of drug administration conducted in a highly complex public university hospital with 263 beds in the Northeast region of Brazil. This study was conducted in two units: a medical (21 beds) and a surgical (23 beds) clinic; both clinics have patients with acute diseases, mostly with more than one chronic disease, who use prescribed drugs from several pharmacological groups. The medical clinic unit admits patients from neurology, neurosurgery, and orthopedics specialties. In contrast, the surgical clinic unit admits female patients from the specialties of gynecology, plastic surgery, urology, and otorhinolaryngology.

In the nursing care routine of this hospital, nursing technicians are responsible for both the preparation and administration of medications, except chemotherapy drugs, and for bathing, feeding, and providing basic care to patients. In turn, the nurses are responsible for supervising the technicians, performing administrative duties, and applying bandages and catheters, among other duties.

### Medication distribution system

Medicines are dispensed per patient, accompanied by a copy of the medical prescription, over a 24-h period, via a distribution system for individualized doses. The pharmacist evaluates the prescription regarding the indication, dose, route of administration, frequency of administration, and drug interactions. After validating the prescription, pharmacy assistants prepare the medication doses per patient, which are checked by the pharmacists before dispensing. Subsequently, the medications are distributed to the units, where they are received and checked by the nursing technicians. After checking, the medications are prepared in the wards and administered to patients by the nursing team at predetermined times, using the original prescription to record the administered times. Unused doses are returned to the pharmacy, using medication carts.

### Data collection

Data were collected in February 2019 by two researchers with at least 2 years of experience in the hospital’s pharmacy, who were trained in the direct observation method by the main researcher. A pilot study was conducted to habituate the professionals to the direct observation method and refine the data collection instrument. Here, the preparation and administration of 23 doses by the same nursing technician was simultaneously observed by the two researchers, and then an agreement between them was determined by calculating the Kappa index (0.8), which was considered satisfactory. Nurses and technicians were informed that the study aimed at improving the hospital’s medication distribution system, however, the objective of identifying MAEs was not explained.

A form for data collection was developed (Additional file 1: Appendix I), containing fields to fill in the following information: date, name of the observer, time of the round, shift, census of the unit, name of the patient (subsequently coded), medication, dose (amount administered), pharmaceutical form, route of administration, time and technique of administration, interruptions, and number of beds per technician.

The observation period in each unit was 15 consecutive days, and the observations were performed 24 h a day in three shifts: morning (7:00 am–1:00 pm), afternoon (1:00 pm–7:00 pm) and night (7:00 pm–07:00 am). The ratios of nursing technician per bed were 5:1 and 4:1 in the medical and surgical clinic units, respectively.

The field researcher was always present in the unit 2 h before the starting times of each medication administration established by hospital standards, until the end of the procedures performed by nurses, thereby witnessing the entire preparation and administration processes of these doses by the nurses.

Some measures were taken to prevent already known biases that may adversely affect the validity of the study. During the data collection process, the observer was not obstructive, neither did they make judgments about the nurse/technician’s work, thereby maintaining a distance that allowed the performance observation of the procedure without disturbing the observed professional (nurse/technician). All selected researchers were experienced pharmacists who were trained in the direct observation method by the main researcher. For 2 days, a test was conducted to familiarize the research team with the clinical unit and identify the need to improve the data collection form, which attested the reliability of the tool.

The data collection process comprised the following steps:

1. Two researchers accompanied the nurses/technicians in the rounds of medication administration, observing the preparation and administration steps, with each researcher in one of the selected units.
2. Each field researcher took notes on the data collection form, detailing the actions of the nurse/technician at the time of medication preparation and administration (medication, dose administered, route of administration, time, etc.).
3. After each round, the observer and main researcher prepared their independent prescription copies of the patients involved. Each dose observed was compared with the dose prescribed by the physician, and in the case of discrepancy, the error was described and categorized.

After comparing all observed doses, each researcher determined whether an additional medication should have been administered during the observation period, based on the medical prescription. If yes, the researcher recorded this as a “dose omission”, unless there was a valid reason for non-administration (e.g., patient discharge, death, or transfer). All collected data were reviewed by the researcher to ensure data validity and reliability. All the obtained information was forwarded to the main investigator, who independently determined administration errors by comparing each dose from the data collection forms with the copies of prescriptions used by the field researchers. Only the errors confirmed by the main researcher were ultimately reported.

### Ethical considerations

The study was submitted to the Research Ethics Committee of the University Medical Center (Professor Edgard Santos) and was approved under opinion number 3,102,570/2019. For ethical reasons, if any error with harmful potential was identified by the field researcher, the researcher would intervene, thereby preventing administration and averting the occurrence of harm to the patient.

### Definitions

A medication administration error has been defined as “the administration of a dose of medication that differs from the prescription, as written in the medical record, or from standard hospital policy and procedures” [8, 9].

Accordingly, drug administration errors were classified into the following categories: omission, non-prescribed dose, extra dose, wrong dose, wrong route, wrong pharmaceutical form, wrong technique, and time error (Additional file 1: Appendix II).

The drugs administered were classified according to the *Anatomical-Therapeutical-Chemical Classification* (ATCC) of the World Health Organization.

### Factors contributing to the occurrence of MAE

The following variables were considered to assess the risk factors that may contribute to the occurrence of errors: type of unit, number of patients under the care of the health professional, ATCC, interruptions during medication preparation and administration, day of the week or shift or time/round, route of administration (oral, intravenous (IV), subcutaneous, inhalation, nasoenteric catheter), and IV or non-IV.

### Data analysis

The analysis solely considered the doses prepared and administered in the presence of the observer and the doses not administered during the observation period. The doses prepared and administered by nursing students or assistants under training were not considered, nor were those prescribed illegibly, rejected by the patient, administered by the patient themself, or referring to missing medications.

### Error rate calculation

The basic measurement unit used was the “total opportunity of error (TOE)”, which is defined as all administered and omitted doses, corresponding to the denominator of Eq. 1 (the total error rate was calculated by dividing the number of doses with one or more errors by the TOE). Similarly, the rate of each type of error was calculated by dividing the number of errors of that particular type by the sum of the administered and omitted doses:

1 $${\text{Error rate}} = \left[ {\left( {{\text{Number of errors }}\left( { < {\text{1 error}}/{\text{dose}}} \right)} \right)/\left( {{\text{Number of administered doses }} + {\text{ omitted doses}}} \right)} \right]*{1}00.$$

**Equation** **1** **. Calculation of the general error rate**

The following rates were calculated:

- Total error rate.
- Error rate by category types (omission, non-prescribed dose, etc.).

### Sample size

To determine the rate of administration errors (% of success in the population (incidence) the period of time (day) was used as a reference). The sample size was calculated using the rationale of the previous study and based on the error rate of estimated medication (10%) from a pilot study of 50 observations [10]. A sample size of 139 doses would be required to achieve 80% power in a two-sided test with a 5% significance level. Dropout rate of 10% (data not valid), approximately 153 doses were considered for the study.

### Statistical analyses

Data were analyzed using descriptive statistics. Errors were scaled by simple frequency per category. For each error category, the mean and standard deviation of the error rate were determined. The SPSS software for Windows, version 26, was employed. Initially, an analysis of the agreement between the two observers was conducted using the Kappa index. All variables were examined in univariate and multivariate formats. The level of significance was set at 5%. The *odds ratio* (OR) was calculated with 95% confidence intervals (CI) and the authors adopted the Chi-square and Mann–Whitney tests for associations. The data were tabulated according to the relative frequency of the types of errors and CI. Subsequently, error rates were compared between the medical and surgical clinics, thereby estimating the significance level of the difference between the percentages (rates) for each clinic.

Multivariate analysis was performed to explore the possible factors associated with errors. The independent variables included characteristics of the medication (ATCC and administration route); characteristics related to administration (day of the week, round of medication, shift and time of administration and interruptions during preparation and administration); characteristics of the observed professional (years of experience and number of patients under the professional’s care); and type of ward.

## Results

The administration of 561 doses of drugs in two in-patient wards of a university hospital was analyzed. In total, 400 doses (71.3%) were administered in the surgical clinic unit and 161 (28.7%) in a medical clinic unit. The total medication administration error rate was 36.2% (95% CI 32.3–40.2). Excluding wrong time errors, the total error rate was 25.1% (95% CI 24.3–32.4). In general, 203 errors were identified. Considering both wards, the most frequent errors were technique (15.5%), time (11.1%), dose (4.8%), and omission (4.5%) errors. Extra dose (0.7%), pharmaceutical form (0.5%), non-prescribed dose (0.4%), and route of administration (0.2%) errors were significantly less frequent (Table 1). When comparing the total medication administration error rates between the two in-patient units, it was observed that the clinical unit had 1.7 times more errors than the surgical unit (Table 2).

**Table 1:** Number and frequency (%) of MAEs, according to type, in two in-patient units

Error category	*n*	%
Error of administration technique	87	15.5
Time error	62	11.1
Wrong dose	27	4.8
Error of omission	25	4.5
Extra dose	4	0.7
Pharmaceutical form error	3	0.5
Non-prescribed dose	2	0.4
Administration route error	1	0.2
Total	203	100.0

University Hospital Edgard Santos, Salvador, BA, Brazil. September 2019

**Table 2:** Number (*N*) and proportion (%) of medication administration errors, according to the in-patient unit

Unit type	Surgical		Clinic		Total	
Error	*N*	%	*N*	%	*N*	%
Yes	120	30.0*	83	51.6*	203	36.2
No	280	70.0	78	48.4	358	63.8
Total	400	100.0	161	100.0	561	100.0

University Hospital Edgard Santos, Salvador, Bahia, Brazil. September 2019

^*^p < 0.001

Comparing the types of administration errors, there were statistically significant differences between the two in-patient units regarding the total error rate. Technique errors were four times more frequent in the clinical unit (Table 3).

**Table 3:** Number and frequency of MAEs, according to the type of error and in-patient unit

Error category	Surgical unit		Clinical unit		Total	
	*N*	%	*N*	%	*N*	%
Technique error	34/366	8.5*	53/108	32.9*	87/561	15.5
Time error	45/400	11.3	17/161	10.6	62/561	11.1
Extra dose	2/398	0.5	2/159	1.2	4/561	0.7
Pharmaceutical form error	3/397	0.8	–/161	–	3/561	0.5
Non-prescribed dose	2/398	0.5	–/161	–	2/561	0.4
Wrong route	–/400	–	1/160	0.6	1/561	0.2
Wrong dose	20/380	5.0	7/154	4.3	27/561	4.8
Dose omission	18/382	4.5	7/154	4.3	25/561	4.5
Total	120/400	30.0*	83/161	51.6*	203/561	36.2

University Hospital Edgard Santos, Salvador, Bahia, Brazil. September 2019

^*^p < 0.001

**Fig. 1:**
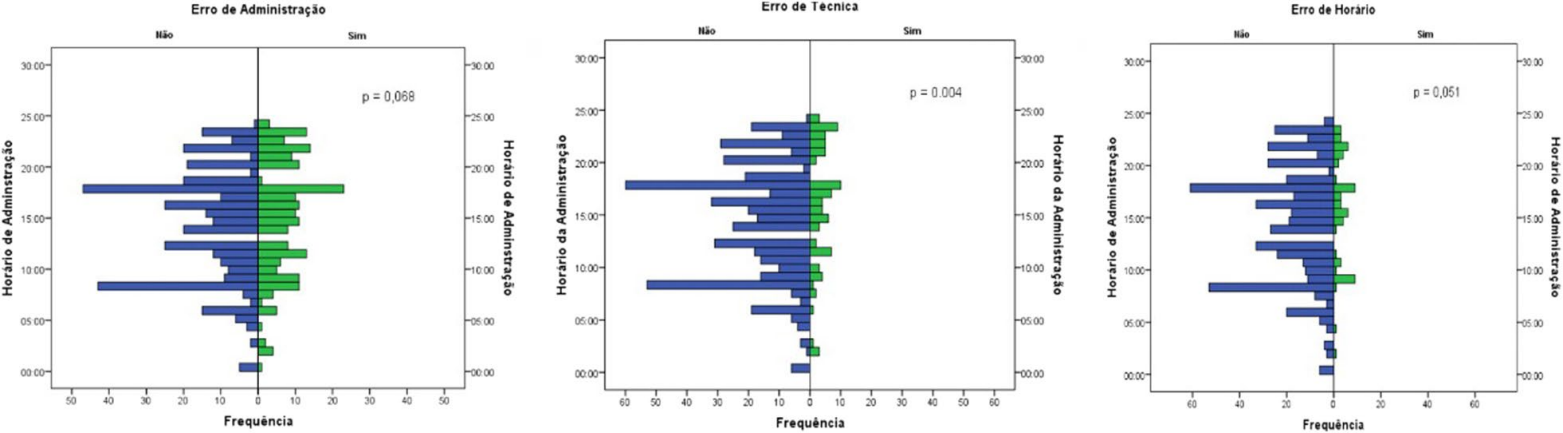
Distribution of MAEs, according to the time of dose administration

Considering the most frequently observed error categories in this study, we selected some examples of MAE (Table 4). When analyzing the occurrence distribution of MAEs, according to the time of dose administration, it was observed that technique errors were more frequent between 4:18 pm and 5:44 pm, while time errors occurred mostly between 2:27 pm and 5:28 pm. It can be observed that the two most frequent errors occurred predominantly in the afternoon shift. Hence, it was determined that the two most frequent errors occurred predominantly in the afternoon shift (Fig. 1).

**Table 4:** Examples of MAEs, according to the most frequent categories

Type of MAE	Examples
Technique error	Vancomycin (1 g) was prescribed to be given for 2 h by intravenous infusion and was administered for 40 min
Time error	Clonidine (0.2 mg) was prescribed for 8:00 pm and was administered at 9:10 pm
Error of omission	4 IU of regular insulin was prescribed for HGT = 190–250. The patient had HGT = 191
Dose error	Atenolol (50 mg) was prescribed and a dose of 25 mg was administered
Error of non-prescribed dose	Codeine (30 mg, without association) was prescribed and codeine (30 mg) + paracetamol (500 mg) was administered
Route error	Oral metoclopramide (10 mg) was prescribed and intravenous metoclopramide (10 mg) was administered

University Hospital Edgard Santos, Salvador, Bahia, Brazil. September 2019

The ATCC categories associated with higher frequencies of errors were digestive system and metabolism (category A) medicines, anti-infective medicines for systemic use (category J), and medicines of the nervous system (category N). These categories concentrated approximately 40% of the MAEs observed in all administered doses. Higher error frequencies were observed in the administration of drugs for the musculoskeletal system (category M) (50.0%) and for the sense organs (category S) (42.9%); however, the number of observations was negligible. Errors were observed in a quarter of the administrations for medicines of the blood and hematopoietic organs (category B) and those of the cardiovascular system (category C) (Table 5).

**Table 5:** Number (*N*) and frequency (%) of MAEs, according to the pharmacological class of the administered medication (ATCC, WHO, 2020)

ATCC classification of medicinal products	Error				Total	
	No		Yes			
	*N*	%	*N*	%	*N*	%
M—musculoskeletal system	3	50.0	3	50.0	6	100.0
S—sense organs	4	57.1	3	42.9	7	100.0
A—digestive system and metabolism	87	58.4	62	41.6	149	100.0
J—general anti-infectives for systemic use	44	58.7	31	41.3	75	100.0
N—nervous system	87	58.8	61	41.2	148	100.0
C—cardiovascular system	45	73.8	16	26.2	61	100.0
B—blood and hematopoietic organs	57	74.0	20	26.0	77	100.0
R—respiratory system	8	88.9	1	11.1	9	100.0
H—systemic hormonal preparations*	16	94.1	1	5.9	17	100.0
Other	7	58.3	5	41.7	12	100.0
Total	358	63.8	203	36.2	561	100.0

^*^Excluding sex hormones and insulin

### Factors associated with the occurrence of MAEs

The independent variables analyzed to assess the existence of risk factors for the occurrence of MAEs are presented in Table 6. The administration route and ATCC classification were risk factors with statistical significance for the occurrence of any error (*p* < 0.05); however, considering the measure of association and its CI, there are no differences between groups. The occurrence of interruptions was identified as a statistically significant factor for the occurrence of technique and time errors, 1.6 times more likely to trigger technique error and 2 times more likely to cause time error. A 1.8 times greater chance of technique error was identified when a technician was responsible for more than 4 beds.

**Table 6:** Risk factors associated with the occurrence of any error, technique errors, and time errors

Risk factors	*n* (%)	Some error		Technique error		Time error	
		*n* (%)	OR (95%CI)	*n* (%)	OR (95%CI)	*n* (%)	OR (95%CI)
Administration hours			*p* = 0.761		*p* = 0.565		*p* = 0.859
Day	338 (60.2)	124 (36.7)	1.036 (0.826–1.298)	50 (14.8)	1	38 (11.2)	1.045 (0.645–1.692)
Night	223 (39.8)	79 (35.4)	1	37 (16.6)	1.122 (0.759–1.657)	24 (10.8)	1
Administration shift			*p* = 0.881		*p* = 0.323		*p* = 0.783
Morning	144 (25.7)	51 (25.1)	0.998 (0.753–1.321)	17 (19.5)	0.737 (0.432–1.259)	15 (24.2)	1.003 (0.544–1.846)
Afternoon	186 (33.2)	70 (34.5)	1.060 (0.822–1.386)	33 (37.9)	1.108 (0.722–1.699)	23 (37.1)	1.190 (0.695–2.039)
Night	231 (41.2)	82 (40.4)	1	37 (42.5)	1	24 (38.7)	1
Day			*p* = 0.346		*p* = 0.977		*p* = 0.170
Monday	66 (11.8)	24 (11.8)	1	11 (12.6)	1	6 (9.7)	1
Tuesday	148 (26.4)	60 (29.6)	1.115 (0.767–1.621)	23 (26.4)	0.932 (0.483–1.799)	20 (32.3)	1.486 (0.626–3.530)
Wednesday	98 (17.5)	33 (16.3)	0.926 (0.607–1.414)	14 (16.1)	0.857 (0.415–1.770)	8 (12.9)	0.898 (0.327–2.469)
Thursday	131 (23.4)	50 (24.6)	1.050 (0.713–1.545)	18 (20.7)	0.824 (0.414–1.642)	17 (27.4)	1.427 (0.591–3.450)
Friday	59 (10.5)	17 (8.4)	0.792 (0.475–1.323)	11 (12.6)	1.119 (0.524–2.388)	4 (6.5)	0.746 (0.221–2.515)
Saturday	32 (5.7)	7 (3.4)	0.602 (0.290–1.246)	6 (6.9)	1.125 (0.457–2.769)	1 (1.6)	0.344 (0.043–2.736)
Sunday	27 (4.8)	12 (5.9)	1.222 (0.720–2.074)	4 (4.6)	0.889 (0.310–2.548)	6 (9.7)	2.444 (0.865–6.911)
Route of administration			***p* < 0.001**				
Intravenous	208 (37.1)	127 (61.1)	2.290 (0.982–5.337)	85 (40.9)	–	26 (12.5)	–
Oral	275 (49.0)	54 (19.6)	0.736 (0.308–1.762)	2 (0.7)	–	30 (10.9)	–
Subcutaneous	63 (11.2)	18 (28.6)	1.071 (0.425–2.704)	0 (0.0)	–	6 (9.5)	–
Other	15 (2.7)	4 (26.7)	1	0 (0.0)	–	0 (0.0)	–
ATCC classification			***p* = 0.023**			*p* = 0.463	
A	149 (26.6)	62 (30.5)	1.632 (0.898–2.968)	27 (31)	–	18 (29)	1.540 (0.521–4.551)
B	77 (13.7)	20 (9.9)	1.019 (0.507–2.048)	7 (8.0)	–	8 (12.9)	1.325 (0.399–4.399)
C	61 (10.9)	16 (7.9)	1.029 (0.495–2.139)	4 (4.6)	–	5 (8.1)	1.045 (0.721–6.778)
J	75 (13.4)	31 (15.6)	1.622 (0.849–3.099)	18 (20.7)	–	13 (21)	2.210 (0.721–6.6778)
N	148 (26.4)	61 (30)	1.617 (0.889–2.943)	31 (35.6)	–	14 (22.6)	1.206 (0.397–3.3664)
Other	51 (9.1)	13 (6.4)	1	0 (0)	–	4 (6.5)	1
Interruptions			***p* = 0.001**		***p* = 0.04**		***p* = 0.01**
Yes	83 (14.8)	44 (53)	1.594 (1.255–2.024)	19 (22.9)	1.609 (1.024–2.529)	16 (19.3)	2.003 (1.192–3.366)
No	478 (85.2)	159 (33.3)	1	68 (14.2)	1	46 (9.6)	1
Beds per technician			*p* = 0.228		***p* = 0.023**		*p* = 0.908
< 4	104 (18.5)	34 (16.7)	1	13 (14.9)	1	12 (19.4)	1
4	322 (57.4)	112 (55.2)	1.064 (0.77–1.456)	43 (49.4)	1.068 (0.598–1.907)	34 (54.8)	0.915 (0.492–1.701)
> 4	135 (24.1)	57 (28.1)	1.292 (0.920–1.813)	31 (35.6)	1.837 (1.013–3.331)	16 (25.8)	1.027 (0.508–2.076)

Values are expressed as simple frequencies and percentages

Bold values are statistically significant

### Technique and time errors

Considering the time errors, the most important factors associated were the technicians’ *interruptions* during the medication preparation and administration processes. Regarding the technique errors, the most important factors were *the route of administration, interruptions, and workload (ratio of number of patients/assisted beds per technician).*

### ATCC

Medicines of therapeutic group A (digestive system and metabolism) and N (nervous system) were the most related to the occurrence of errors, with proportions of 30.5% and 30%, respectively (*p* = 0.023).

### Route of administration

Intravenous administration was 5.71 times more associated with errors than non-intravenous administration (Table 7). Considering the in-patient unit, there was a 1.6 times higher risk of error in intravenous administration in surgical wards than in medical clinic wards (Table 8).

**Table 7:** Number (*N*) and frequency (%) of MAEs, according to the route (intravenous and non-intravenous administration)

Administration error	No		Yes		Total	
Route of administration	*N*	%	*N*	%	*N*	%
Intravenous	277	74.4	76	37.4	353	62.9
Not intravenous	81	22.6	127	62.6	208	37.1
Total	358	100	203	100	561	100.0

*p* < 0.05; direct observation 5.71 (95% CI 3.9–8.3) risk for intravenous route compared to non-intravenous route

**Table 8:** Number (*N*) and frequency (%) of MAEs, according to the route (intravenous and non-intravenous administration) and in-patient unit

Administration route	Surgical		Clinic		Total	
	*N*	%	*N*	%	*N*	%
Intravenous	59	49.2	17	20.5	76	37.4
Not intravenous	61	50.8	66	79.5	127	62.6
Total	120	100.0	83	100.0	203	100.0

*p* < 0.05; OD = 1.61 (95% CI 3.9–8.3) of intravenous route risk in surgical ward when compared to clinical

## Discussion

This study, conducted in two in-patient units of a University Hospital, identified a total rate of MAEs of 36.2% (203/561), which is relatively high, even when time errors (25.1%) are excluded. This finding is similar to those described in studies carried out in Brazil and other countries [4, 11–14].

However, it should be noted that there is a wide variation in the rates of MAE deduced from both international (8.6% to 28.3%) and national (9% to 64%) studies. In studies conducted in Latin America, including Brazil, using the same methodology, the average rate of MAE was approximately 30% [7, 15–18], which is three times the average rates in developed countries (10%) [14, 19].

The large variation identified in the studies may be related to methodological factors such as different definitions and/or adopted MAE classification, including the approach employed in calculating the error rates, as well as the inclusion and exclusion criteria adopted [20].

Technique, time, dose, and omission errors occurred more frequently than other errors. Most technique errors were related to injectable drugs whose administration speeds were inadequate when compared to the permissible speed rate determined by the hospital dilution manual [21]. It was observed that technique errors occurred 3.5 times more often in the clinical medicine unit than in the surgical unit. Although the university hospital has a dilution manual and a Patient Safety Program, there was a substantially high rate of technique errors when compared to other national studies [7]. Technique errors, especially in the case of intravenously administered doses, have a significantly high potential to cause harm. Taxis and Barber determined the lack of training of the nursing team as one of the main causes of errors in intravenous drug administration [14]. These errors may also be associated with the complexity in the preparation and administration of these medications.

The cause of technique errors can be multifactorial, thereby requiring further studies [4, 19, 22]. A study on the evaluability of the Medication Dilution Manual of HUPES determined the need for team training on the proper use of the manual as the main result reported by physicians, pharmacists, nurses, and nursing technicians, which can contribute to minimizing the technique errors, because, as identified in this study, these errors were often related to non-compliance with the recommendations described in the dilution manual [23].

The time error was the second most frequent error, and it occurred significantly more in the afternoon shift, precisely at 14:27 ± 5:28 pm, while technique errors occurred more at 16:18 ± 5:44 pm. The wrong time error is frequently identified in most studies and is usually not severe. However, it may become increasingly serious for some medications, especially those that need to be administered in a very narrow time window to achieve the desired therapeutic result and/or avoid adverse events [4, 11]. In these cases, some institutions specify the medications that are considered critical in terms of administration time, such as those that can cause harm or have a significant negative impact on their therapeutic or pharmacological effect, if they are administered early or late (more or less than 30 min from the scheduled time) [24]. Hence, “potentially dangerous” medications are important because maintaining the therapeutic effect depends on the accuracy of the schedules relative to feeding or the maintenance of plasma levels [25].

The third most frequent error was the dose error, whose rate was 4.8%, occurring both in the administrations of injectable and solid-oral medications. Eight studies conducted in Latin American hospitals determined a huge variation in dose errors, ranging from 1.7 to 50%. It is unclear how this variation can be explained: whether by differences in the concept of dose error or by the inclusion of the administration of extra dose in this same category [7, 15–17, 26–30]. Berdot and collaborators in a meta-analysis deduced an average in dose error rate of 1.4%, three times lower than that identified in this study [19]. Dose errors are crucial, both for treatment effectiveness and patient safety.

The fourth most frequent error was omission, with 4.5% rate on average—less than half the rates identified in other studies that adopted the direct observation method (10% of omitted doses) [7, 13, 15, 19, 31]. Errors of omission are frequent and can cause harm to patients, especially if they involve the intravenous route. The causes and contributing factors of these errors are well known and mostly related to communication problems [32].

### Associated factors

The risk factors associated with the MAEs presented in this study were route of administration, interruptions, workload (number of beds per nursing technician), and drug class (ATCC).

Complexity in preparation and administration is, by itself, a risk factor. Complexity is mainly observed with drugs administered intravenously. In this study, this route of administration had 5.71 times (*p* < 0.05; 95% CI 3.9–8.3) higher risk of error than the non-intravenous route. In the reviewed studies, the rates of administration errors by the intravenous route varied widely, ranging from 1 to 70%. Again, this variation is probably attributed to methodological differences between them. A number of authors have studied intravenous medications alone [33], whereas others have studied both intravenous and non-intravenous medications [14]. In some studies, error rates were determined in both the preparation and administration phases, while in others, rates were solely calculated in one phase [34]. Finally, there were differences in the definitions and classification of errors among the various studies [7, 13–15].

Even considering that these differences make comparisons difficult, research evidence suggest that the intravenous route should be prioritized in hospital strategies to reduce errors with higher potential for causing harm [20]. The hospital where the study was conducted recently published a Procedures Manual for Intravenous Administration; however, a high rate of errors by this route was still observed, particularly in the surgical clinic when compared to the medical clinic. These differences between in-patient units may be associated with their characteristics in terms of patient profiles, with more frequent intravenous administrations in the surgical unit than in the medical clinic unit, including the organization of nursing work, knowledge, and skills of nursing technicians, and already known risk factors for MAEs [22].

The analysis indicated that doses administered by nursing technicians with interruptions during administration had 1.59 times (95% CI 1.255–2.024), 1.61 times (95% CI 1.02–2.53), and 2.00 times (95% CI 1.19–3.37) more route of administration, technique, and time errors, respectively, compared to those administered without interruptions, thereby demonstrating that this risk factor offers a higher occurrence probability of errors, thereby causing harm to patients [33, 35–37].

Few Brazilian studies have explored the risk factors associated with MAEs. A single study demonstrated that the nursing workload generally increases the risk of MAE occurrence, by a factor of 7, which is higher in the case of time errors (8 times). These findings are consistent with those of the international literature [7, 37, 38].

Another important factor is related to the number of assisted beds per professional. In this study, a 1.8 times higher risk of technique errors was determined in cases where there were more than a four-bed distribution per professional, compared to cases in which there were up to 4 beds per professional. The number of patients under the care of a single nursing professional also related to the occurrence of any type of error and time errors, but without statistical significances in these cases. These risk factors were also identified in a study conducted by Grou Volpe et al. [7]. Increased workload was also related to a higher risk of time and preparation errors.

When correlating therapeutic classes to MAEs, it was deduced that the drugs for the digestive system and metabolism (A) and those for the nervous system (N) were the most associated with technique errors, while those of classes A, N, and J (anti-infective of systemic use) were more associated with time errors. In a study conducted at a university hospital in Brasília, Volpe et al. [7] determined that the therapeutic classes most related to time errors were the drugs for the cardiovascular system (C), nervous system (N), and injectable antibiotics (J).

The findings of this study are important because they indicate that the most frequent errors such as technique and time errors are related to the therapeutic class of the medication, interruptions, and route of administration. It is known that the severity of errors is significantly higher when the medication is intravenously administered [1, 7]. Prevention strategies should be aimed at controlling these contributing factors, especially for potentially dangerous drugs and intravenously administered drugs.

Although the university hospital has an active patient safety program, a pharmacy service with clinical pharmacists in in-patient units, and an available medication administration manual (dilution manual), this study still identified a high rate of MAEs, thereby demonstrating the need for further studies focusing on MAEs with higher severity and potential risk of causing harm.

This study made important contributions, as it was the first to calculate the occurrence of MAEs in our hospital. Its results reinforce the need to conduct new studies with the same methodology, to facilitate designing interventions that reduce the current error rate to permissible levels.

Although it followed an internationally validated methodology in identifying MAEs, the present study was solely conducted in two units of a university hospital, which limits the possibilities of comparing and extrapolating results to other healthcare environments. The possible adverse influence of the presence of an observer on the observations was minimized by the training of the observers, who were guided to adopt an ethical and non-obstructive approach.

## Conclusion

The total MAE rate was high, with technique, time, dose, and omission errors being the most frequent, especially in the clinical medicine unit, which agrees with the results of other national and international studies. In addition, it is noteworthy that the highest risk of error was observed in intravenous drug administrations.

Specifically, regarding the hospital studied, these findings indicate the need to develop a safer medication use system that ensures less risk to patients and professionals in the studied environment. In general, although the study was conducted in a single hospital, the details provided by the types of errors and their severity can be beneficial in other contexts, thereby ensuring the adoption of more specific risk minimization strategies.

## Supplementary Information

**Additional file 1. Appendix 1.** Data collection form designed for data collection during medication administration round. **Appendix 2**. Eight categories of medication administration errors.

## Data Availability

The dataset(s) supporting the conclusions of this article is(are) included within the article (and its additional file(s)).
