# Supplementary material for: Observational study on medication administration errors at a University Hospital in Brazil: incidence, nature and associated factors
Source: J Pharm Policy Pract. 2022 Aug 22;15:51. doi: 10.1186/s40545-022-00443-x (PMC9396806; doi:10.1186/s40545-022-00443-x)
Supplement: Supplementary file 1 — Additional file 1. Appendix 1. Data collection form designed for data collection during medication administration round. Appendix 2. Eight categories of medication administration errors. [file 40545_2022_443_MOESM1_ESM.docx]

**Appendix I - Data Collection Form**

| **Date:** | **Observer:** | **Ward census:** |
| --- | --- | --- |
| **Which round?** | **Nurse/auxiliary:** | **Page _____ of _____** |

| **Schedule** | **Sample** | **Pack ID / bed** | **Did you check the ID?** | **Medicines (details)** | **Admin. obs.?** | **Will you sign?** | **Cod.** |
| --- | --- | --- | --- | --- | --- | --- | --- |
|  |  |  |  |  |  |  |  |
|  |  |  |  |  |  |  |  |
|  |  |  |  |  |  |  |  |
|  |  |  |  |  |  |  |  |
|  |  |  |  |  |  |  |  |
|  |  |  |  |  |  |  |  |
|  |  |  |  |  |  |  |  |
|  |  |  |  |  |  |  |  |
|  |  |  |  |  |  |  |  |
|  |  |  |  |  |  |  |  |
|  |  |  |  |  |  |  |  |
|  |  |  |  |  |  |  |  |
|  |  |  |  |  |  |  |  |
|  |  |  |  |  |  |  |  |
|  |  |  |  |  |  |  |  |
|  |  |  |  |  |  |  |  |
|  |  |  |  |  |  |  |  |
|  |  |  |  |  |  |  |  |
|  |  |  |  |  |  |  |  |

**APPENDIX II - CATEGORIES OF MEDICATION ERRORS**

- **Omission:** A dose of medication that has not been administered by the time of the next scheduled dose. Doses according to physician instructions, nurse clinical judgment, or the absence of the patient from the ward, are not included in this category.
- **Unprescribed dose:** The administration of a drug dose that was never prescribed for the patient. It is classified as a wrong drug if drug X was given instead of the prescribed drug Y.
- **Extra dose:** The administration of an additional dose to the prescribed medicine. It includes taking the medicine more times a day than prescribed and taking another dose when the prescription is terminated.
- **Wrong Dose:** Any dose of a correct drug via the correct route, but in a different amount than prescribed (Inappropriate amount or number). For injectable drugs, any dose that is ±10% or more of the correct dose; for any other pharmaceutical form, any dose that is ±17% or more of the correct dose in the observer's judgment. In the judgment of doses, the measurements obtained with devices or appliances usually used in the institution should be considered (graduation in syringes, dosing burette, dropper, etc.)
- **Wrong Route:** The administration of a correct drug via a route or place of administration that differs from the prescription. Administration of a drug via the oral route when the prescription required the intramuscular route. Included in this category is the administration of eye drops to the left eye when it was prescribed to be applied to the right eye.
- **Wrong Pharmaceutical form:** The administration of the correct dose of a drug via the correct route, but not prescribed in a pharmaceutical form, especially when this has been specified. Included in this category is the administration of a slow-release pharmaceutical form when a rapid release form had been prescribed.
- **Wrong Technique:** Exclusion or inadequate performance of a prescribed procedure immediately before the administration of each dose. For example, taking a pulse before administering a beta-blocker.
- **Wrong Time:** the administration of a dose more than 60 min before or after the time scheduled by the nurse. For medications prescribed to be taken before, after, or at food, the administration of a dose more than 30 min before or after food. The time for comparison is the time used by the nurse in the prescription.
